# Optimization of a micro-scale air–liquid-interface model of human proximal airway epithelium for moderate throughput drug screening for SARS-CoV-2

**DOI:** 10.1186/s12931-025-03095-y

**Published:** 2025-01-16

**Authors:** Chandani Sen, Tammy M. Rickabaugh, Arjit Vijey Jeyachandran, Constance Yuen, Maisam Ghannam, Abdo Durra, Adam Aziz, Kristen Castillo, Gustavo Garcia, Arunima Purkayastha, Brandon Han, Felix W. Boulton, Eugene Chekler, Robert Garces, Karen C. Wolff, Laura Riva, Melanie G. Kirkpatrick, Amal Gebara-Lamb, Case W. McNamara, Ulrich A. K. Betz, Vaithilingaraja Arumugaswami, Robert Damoiseaux, Brigitte N. Gomperts

**Affiliations:** 1 Department of Pediatrics, David Geffen School of Medicine, UCLA Children’s Discovery and Innovation Institute, Mattel Children’s Hospital UCLA, UCLA, Los Angeles, CA 90095 USA; 2 Department of Molecular and Medical Pharmacology, University of California, Los Angeles, CA 90095 USA; 3 California Nanosystems Institute, UCLA, Los Angeles, CA 90095 USA; 4 Merck KGaA, Darmstadt, Germany; 5 Jonsson Comprehensive Cancer Center, UCLA, Los Angeles, CA 90095 USA; 6 Eli and Edythe Broad Stem Cell Research Center, UCLA, Los Angeles, CA 90095 USA; 7 Division of Pulmonary and Critical Care Medicine, Department of Medicine, David Geffen School of Medicine, UCLA, Los Angeles, CA 90095 USA; 8 EMD Serono, Billerica, MA 01821 USA; 9 Calibr-Skaggs Institute for Innovative Medicines, 11119 North Torrey Pines Road, La Jolla, CA 92037 USA

**Keywords:** Human mucociliary epithelium, SARS-CoV-2, Respiratory viral infections, High throughput drug screening, Anti-viral screening, Small-molecules, Air–liquid-interface, Heterogeneity, Image quantification, RNA sequencing

## Abstract

**Background:**

Many respiratory viruses attack the airway epithelium and cause a wide spectrum of diseases for which we have limited therapies. To date, a few primary human stem cell-based models of the proximal airway have been reported for drug discovery but scaling them up to a higher throughput platform remains a significant challenge. As a result, most of the drug screening assays for respiratory viruses are performed on commercial cell line-based 2D cultures that provide limited translational ability.

**Methods:**

We optimized a primary human stem cell-based mucociliary airway epithelium model of SARS-CoV-2 infection, in 96-well air–liquid-interface (ALI) format, which is amenable to moderate throughput drug screening. We tested the model against SARS-CoV-2 parental strain (Wuhan) and variants Beta, Delta, and Omicron. We applied this model to screen 2100 compounds from targeted drug libraries using a high throughput-high content image-based quantification method.

**Results:**

The model recapitulated the heterogeneity of infection among patients with SARS-CoV-2 parental strain and variants. While there were heterogeneous responses across variants for host factor targeting compounds, the two direct-acting antivirals we tested, Remdesivir and Paxlovid, showed consistent efficacy in reducing infection across all variants and donors. Using the model, we characterized a new antiviral drug effective against both the parental strain and the Omicron variant.

**Conclusion:**

This study demonstrates that the 96-well ALI model of primary human mucociliary epithelium can recapitulate the heterogeneity of infection among different donors and SARS-CoV-2 variants and can be used for moderate throughput screening. Compounds that target host factors showed variability among patients in response to SARS-CoV-2, while direct-acting antivirals were effective against SARS-CoV-2 despite the heterogeneity of patients tested.

## Background

The Severe Acute Respiratory Syndrome Corona Virus 2 (SARS-CoV-2) pandemic has resulted in more than 7 million deaths worldwide as of June 2024 [1]. It spurred a global effort to develop new drugs and therapies to combat the virus. Monoclonal antibodies and vaccines were rapidly developed within a year of the start of the pandemic. However, there is still an ongoing search for better small molecules for current and evolving SARS-CoV-2 strains with fewer side effects and drug interactions, especially for immunocompromised and unvaccinated patients. The continuing research has focused on understanding and modulating both the host factors [2–5] and viral factors [6–8] influencing the severity of COVID-19.

High throughput drug screening (HTS) of FDA-approved repurposed drug libraries is a preferred strategy for drug discovery because of the compounds’ known safety profile, cost-effectiveness, and time efficiency [9, 10]. There have been many drug screening efforts from the onset of the COVID-19 pandemic, using both computational [11–14] and in vitro methods. The traditional in vitro high throughput screens (HTS) for respiratory viral infections are often performed on 2D cultures of commercial cell lines like Vero E6, Huh7, Huh7.5, Caco2, Calu-3, A549, and several other non-human cell lines. These cell line-based drug screenings lack the highly specialized human mucociliary airway epithelial cell types and the epithelium’s innate immune response.

Air–liquid-interface (ALI) cultures are well-established models that mimic the micro-physiological conditions of the functional mucociliary epithelium and can also be used to study disease pathogenesis [15–17]. ALI cultures can overcome the limitations of traditional 2D cell-line-based cultures as they demonstrate differentiation to the relevant cell types, epithelial barrier function, and an innate immune response. The ALI culture model has demonstrated the mechanisms of SARS-CoV-2 infection of airway cells, the role of exposures (e.g., smoking) on infection severity, and low throughput drug testing [18–23]. Despite ALI cultures having great potential to study drug efficacy for respiratory viral infections scaling up the ALI model to a robust, high-throughput drug screening platform remains a challenge due to the complexity of this culture model [15–19, 22, 23]. Recently, there has been a growing interest in developing higher throughput ALI cultures [24–26]. Still, no reports exist on optimizing human primary cell-based high throughput ALI systems as a drug screening platform for SARS-CoV-2 infection. Also, there have been no studies to examine the heterogeneity between human airway stem cell donors in their response to SARS-CoV-2 infection in ALI cultures, and there is no information about modeling infection severity across SARS-CoV-2 variants.

Therefore, we developed a 96-transwell primary human mucociliary epithelial ALI culture platform with primary SARS-CoV-2 infection and developed a novel image-based quantification method to quantify this infection. We used primary human airway basal stem cells from eight patients with no previous history of lung disease, the parental SARS-CoV-2 strain (Wuhan), and three SARS-CoV-2 variants (Beta, Delta, and Omicron), and three drug libraries to screen 2100 repurposed small molecules. We noticed significant heterogeneity of infection based on donors and SARS-CoV-2 variants. We also found a lack of consistency in the efficacy of drugs targeting host factors. However, when screening with direct-acting antivirals, we found consistent results across all donors and SARS-CoV-2 variants. We used our 96-transwell ALI platform to characterize a new small molecule with antiviral properties and validated its efficacy against the parental strain and the Omicron variant. This 96-transwell primary human mucociliary epithelial ALI culture platform with SARS-CoV-2 infection shows potential for drug screening for respiratory viral infections.

## Methods

### Human tissue procurement

Large airways and bronchial tissues were acquired from three different tissue sources: 1. de-identified normal human donors after lung transplantations at the Ronald Reagan UCLA Medical Center. Tissues were procured under an Institutional Review Board-approved protocol at the David Geffen School of Medicine at UCLA (IRB#21-000390-CR-00003). 2. Normal human bronchial epithelial cells (NHBE) from non-smokers were obtained from Lonza and all samples were de-identified. 3. Deidentified donor lung and trachea samples from the International Institute for the Advancement of Medicine (IIAM) were obtained with institutional approval. De-identified patient information for all samples is shown in Table 1.

**Table 1:** List of donor demographics, tested drug libraries, and SARS-CoV-2 variants

Donor number	Demographics	Drug library	Variant tested
DL1	70, M, B, NS, A	LOPAC	Wuhan
DL2	66, M, C, S, A	LOPAC, New Prestwick	Wuhan, Beta
DL3	59, M, C, NS, A	LOPAC, New Prestwick	Wuhan, Beta, Delta
DL4	79, F, C, S, A	NKIL	Wuhan, Delta
DL5	55, F, C, S, A	NKIL	Delta, Omicron
DL6	51, M, C, NS, NA	All previous hits from LOPAC, New Prestwick and NKIL libraries	Wuhan, Omicron
DL7	67, F, C, NS, A	All previous hits from LOPAC, New Prestwick and NKIL libraries	Wuhan, Omicron
DL8	45, F, C, S, A	All previous hits from LOPAC, New Prestwick and NKIL libraries	Wuhan, Omicron
Pooled DL6-8	As above	Top hits from LOPAC, New Prestwick and NKIL libraries + small molecules from Merck KGaA, Darmstadt, Germany	Omicron, Nanoluc

DL: Donor Lung samples; M: male; F: female; B: Black; C: Caucasian; S: smoker; A: alcohol use; NS: nonsmoker; NA: no alcohol use

### ABSC isolation

Human ABSCs were isolated using our laboratory's previously published method [19, 27–29]. All steps were performed with the trachea in cold PBS with antimicrobials. Proximal cartilaginous airways were dissected, cleaned, and incubated in 50 U/ml dispase for 45 min at room temperature. Tissues were then incubated in 0.5 mg/ml DNase for another 45 min at room temperature. The epithelium was stripped and scraped with a cell-scraper and incubated in 0.25% Trypsin–EDTA for 30 min, shaking at 37 °C to generate a single cell suspension. Isolated cells were passed through a series of strainers, 500 μm, 300 μm, and 100 μm strainer, and either plated directly (Passage 0 (P0)) for Air Liquid Interface (ALI) cultures or expanded.

For expansion, the isolated ABSCs were seeded in 0.5 mg/ml collagen IV pre-coated T75 flasks in ‘PneumaCult™-Ex Plus Medium (STEMCELL Technologies)’ at a seeding density of 1 × 10^6^ until they reached 80–90% confluency. The cells were then dissociated from the flask with StemPro Accutase (Thermofisher Scientific), centrifuged at 300 × *g* for 5 min, and the cell pellet was collected. After dissolving into 1 ml of expansion media and counting, the cells were split into a 1:3–1:5 ratio and re-seeded in pre-coated flasks. This process was repeated up to Passage 2 (P2) and then plated for ALI cultures.

### 96-TW ALI cultures

96-TW with 0.4 μm pore polyester membrane inserts (Corning 7369) were coated with 0.5 mg/ml collagen type IV and allowed to air dry overnight inside a biosafety cabinet. ABSCs (P0-P2) were seeded at 8000–10,000 cells per well onto collagen-coated transwells in PneumaCult™-Ex Plus Medium (STEMCELL Technologies), supplemented with Primocin (Invivogen), 1X Penicillin–Streptomycin-Neomycin (PSN) Antibiotic Mixture (Thermo Fisher Scientific). ABSCs were allowed to grow in the submerged phase with 180 μl media in the basal chamber and 75 μl media in the apical chamber until they were 80–100% confluent and tight cell junctions were formed (5–7 days). ALI cultures were then established via airlifting, and cells were cultured with 180 μl PneumaCult™-ALI Medium (STEMCELL technologies) only in the basal chamber for 21 days until viral infection. Media was changed every other day, and cultures were maintained at 37 °C with 5% CO_2_. In the case of drug testing, drugs were added at their required concentration in the basal chamber media 24 h before viral infection.

### SARS-CoV-2 infection

SARS-CoV-2 strains including Isolate USA-WA1/2020 (Parental, BEI Resources NR-52281) at a low MOI (0.1), followed by the Isolate hCoV-19/USA/MD-HP01542/2021 (Beta, BEI Resources NR-55282), Isolate hCoV-19/USA/MD-HP05647/2021 (Delta, BEI Resources NR-55672), and Isolate hCoV-19/USA/MD-HP20874/2021 (Omicron, BEI Resources NR-56461) were obtained from Biodefense and Emerging Infectious (BEI) Resources of the National Institute of Allergy and Infectious Diseases (NIAID). All the propagated strains used in this study are sequence-verified. All the studies involving live viruses were conducted in the UCLA BSL3 high-containment facility with appropriate institutional biosafety approvals. SARS-CoV-2 was passaged once in Vero-E6 cells, and viral stocks were aliquoted and stored at − 80 °C. Virus titer was measured in Vero-E6 cells by TCID_50_ assay [20, 30].

ALI cultures on the apical chamber of transwell inserts were infected with SARS-CoV-2 viral inoculum (MOI of 0.1; 100 µl/well) prepared in PneumaCult™-ALI Medium (STEMCELL technologies). The basal chamber of the transwell contained 180 µl of ALI media. For mock infection, ALI media (30 µl/well) alone was added. The inoculated plates were incubated for 2 h at 37 °C with 5% CO_2_. At the end of incubation, the inoculum was removed from the apical chamber. At selected time points, bright field microscopy obtained live cell images to detect cytopathic effect (CPE) in SARS-CoV-2 infected cells, indicating viral replication and associated cell injury. At 72 h post-infection (hpi), ALI cultures were fixed in 4% paraformaldehyde followed by immunofluorescence (IF) analysis.

### Nanoluciferase

We performed a Nano-Glo Luciferase Assay System as indicated by the manufacturer to assess luciferase activity's presence. ALI Cultures were pretreated with drug compounds for 24 h. Pretreated ALI cultures were then infected with SARS-Related Coronavirus 2, Isolate USA-WA1/2020 (icSARS-CoV-2-nLuc) (BEI Resources NR-54003) After 48 hpi a working reagent luciferin (100 μL) was added to the cells and incubated for at least 3 min at room temperature. Thereafter, the luminescence of each condition was measured and recorded using triplicate values. The percent viability for each compound was calculated based on the treated cells of the vehicle (water or DMSO).

### Immunofluorescence and confocal imaging

**Fig. 1:**
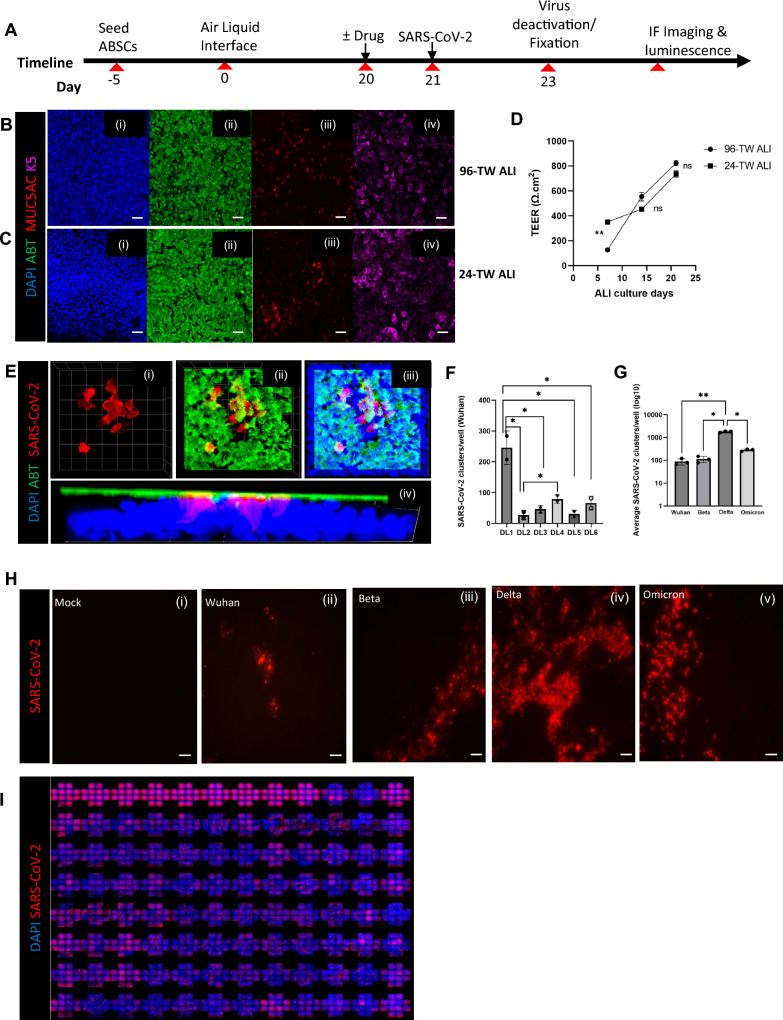
Development of a human primary mucociliary airway stem cell-based SARS-CoV-2 infection model. **A** Schematic of the experimental timeline. **B** 96-TW ALI and **C** 24-TW ALI with immunofluorescent (IF) staining showing (i) Nuclei (DAPI), (ii) Ciliated cells (AβT), (iii) Mucus cells (MUC5AC), (iv) Basal stem cells (K5). Scale bar = 50 µm. **D** TEER values of 24-and 96-TW ALI. P values are calculated from technical replicates using Student’s t-test. **P < 0.01. **E** Confocal imaging (magnification: 63X) of IF staining showing a typical SARS-CoV-2 (Omicron) cluster of infection in the 96-TW ALI model. (i) SARS-CoV-2 infection (Nucleocapsid antibody). (ii) Ciliated cells (AβT) with SARS-CoV-2 infection. (iii) Ciliated cells (AβT) with SARS-CoV-2 and DAPI. (iv) Orthogonal projection of IF staining of the infected 96-TW ALI culture. **F** Quantification of the degree of infection among different human donors. DL: Donor Lung samples. P values are calculated from technical replicates using a one-way ANOVA test. *P < 0.05. **G** Quantification of the average degree of infection among different SARS-CoV-2 Variants. P values are calculated from technical replicates using one-way ANOVA test. **P < 0.01, *P < 0.05. **H** Variability of the degree of infection by IF staining for SARS-CoV-2 in (i) Mock (no infection), (ii) Wuhan (Spike antibody), (iii) Beta (Spike antibody), (iv) Delta (Nucleocapsid antibody), (v) Omicron (Nucleocapsid antibody). Scale bar = 50 µm. **I** Representative HTS images of SARS-CoV-2 infection (Nucleocapsid antibody) with 96-TW ALI model

Post fixation with 4% PFA, the ALI cultures were washed 3 times with Tris-Buffered Saline and Tween-20 (TBST) followed by permeabilization with 0.5% Triton-X for 10 min. ALIs were then blocked using serum-free protein block (Dako X090930) for one hour at room temperature and overnight for primary antibody incubation. Based on preliminary trials, we optimized different SARS-CoV-2 antibodies for different strains of SARS-CoV-2 virus. SARS-CoV [BEI Resources: NR-10361 Polyclonal Anti-SARS Coronavirus (antiserum, Guinea Pig)] for Parental (Wuhan) and Beta strains; SARS-CoV-2 [(2019-nCoV) Spike S1 Antibody, Rabbit Mab] for Parental (Wuhan), Beta and Delta strains, and SARS-CoV-2 nucleocapsid antibody (GeneTex, GTX135357) for the Delta and Omicron strains. After several washes with TBST, secondary antibodies were incubated on samples for 1 h in darkness, washed, and used for confocal imaging. High-throughput, high-content confocal images were obtained using ImageXpress (Molecular Devices) with a 20X objective, and number of infection clusters per well was quantified by the developed algorithm. To compare ALI differentiation in 24- and 96-TW (Fig. 1), images were captured on Zeiss LSM 880 confocal microscopes.

### Image quantification

Plates were imaged using a Molecular Devices Confocal Imager at 20 × resolution with an extra-long working distance (ELWD) objective (n/a = 0.45) with at least 14 fields per well on an Image Xpress Confocal (Molecular Devices) while avoiding the corners of the well. Each site in each well was individually focused by images for the DAPI channel and pictures obtained for both the DAPI and TRITC channels. Images were streamed in real-time into a SQL-managed instance of MetaXpress Software (version 6.5) with a dedicated database and file server, respectively. The image analysis workstation used for data analysis ran ImageXpress equipped with Custom Module Editor. The DAPI channel was utilized to ensure an even cell layer without structural defects and to identify compounds with gross toxicity effects via visual inspection. We used an adaptive thresholding approach to detect clusters of cells infected with SARS-CoV-2. Objects ranging from 21.9 to 250 microns with a brightness of 4500 greyscales over a local background were scored as positive, and the number of infected cell clusters and average intensity were recorded. The average number of infected cell clusters was determined for each well as the mean of all sites for each well.

### Bulk RNAseq analysis

Cells from a 24-well transwell ALI were collected in TRIzol and RNA was extracted using the Zymo Direct-zol RNA Miniprep kit (item number R2050) according to the manufacturer’s instructions. The RNA concentrations were measured on a NanoDrop ND-1000 spectrophotometer. It was then analyzed by RNA-Seq at the Technology Center for Genomics and Bioinformatics (TCGB) core at UCLA.

Illumina libraries for RNA-Seq were prepared using the KAPA RNA Hyper + RiboErase HMR Kit according to the manufacturer’s instructions. Briefly, the workflow consisted of rRNA depletion by hybridizing complementary DNA oligonucleotides, followed by RNase H and DNase treatment. The next steps included mRNA enrichment and fragmentation, first-strand cDNA synthesis using random priming followed by second-strand synthesis converting cDNA: RNA hybrid to double-stranded cDNA (dscDNA), and incorporating dUTP into the second cDNA strand. cDNA generation was followed by end repair to generate blunt ends, A-tailing, adaptor ligation, and PCR amplification. Different adaptor barcodes were used for multiplexing samples in one lane. Sequencing was performed on Illumina HiSeq3000 for a single-end 1 × 50 run. A data quality check was performed using the Illumina SAV. Demultiplexing was performed with Illumina Bcl2fastq v2.19.1.403 software. RNAseq reads were mapped by STAR 2.7.9a [31] and read counts per gene were quantified using the human genome GRCh38.104. Normalization was performed using Partek Flow software [32] and read counts were normalized by CPM + 1.0E−4. For comparison between samples, read counts for each gene were normalized to mock treatment or virus-only treatment, respectively and the top 100 up or downregulated genes were further analyzed utilizing the NIH Database for Annotation, Visualization, and Integrated Discovery (DAVID) to calculate enrichment scores for functional gene clusters and calculate p-values and Benjamini–Hochberg corrected p-values.

### Z’ calculation and statistical analysis

Z’ (Z-factor) for 96-well ALI was calculated following the method described by Zhang et al. [33] as described below:

$${Z}{\prime}=1-\frac{3\times (\text{standard\, deviation\, of\, infection\, wells}+\text{standard\, deviation\, of\, Mock\, wells}) }{\left|mean\, of\, infection\, wells-mean\, of\, mock\, wells\right|}$$

Mean and standard deviation were calculated on infection only (no drug added) and mock (no infection) wells (n > 3) for each SARS-CoV-2 variant.

Student’s t-test was used to determine the statistical significance of TEER value between 96-TW ALI and 24-TW ALI groups. ANOVA was used to compare the number of infection clusters across different donors and different variants. Significance was defined as p < 0.05. Statistical details of experiments can be found in the Figure legends.

### In vivo mouse studies of SARS-CoV-2 infection

In vivo experiments were performed at Vibiosphen. At D0, the mice (B6.Cg-Tg(K18-ACE2)2Prlmn/J males, 7-week-old) were pre-treated by oral gavage 1 h before infection (100 mg/kg compound in 0.5% Methocel/0.25% Tween-20/water). At D0 + 1 h, the mice were infected with 25 µL of DMEM containing SARS-Cov-2 (1 × 104 PFU/mouse) parental strain through the intranasal route. From D0 to D5 mice were treated by oral gavage twice daily. The pause between treatments was set to 12 h. At D1, D3, and D6, 5 mice from each group were euthanized by cervical dislocation for collection of lung tissue and virus quantification.

Lung tissues (up to 30 mg) were homogenized in RLT buffer, and RNA was extracted using the RNeasy kit (Qiagen) according to the manufacturer's instructions. Viral RNA was detected by qRT-PCR.

## Results

### 96-transwell air–liquid-interface (96-TW ALI) model of the human mucociliary airway for drug screening demonstrates the heterogeneity of infection among different airway donors and SARS-CoV-2 variants

We previously showed that the live SARS-CoV-2 virus can infect the 24-well transwell ALI (24-TW ALI) model of human mucociliary epithelium [19]. Since the 24-TW ALI model is sub-optimal for high throughput screening, we optimized a 96-TW ALI for screening purposes. The detailed protocol of 96-TW ALI generation is described in the methods section and the general timeline is shown in Fig. 1A. Briefly, we isolated airway basal stem cells (ABSC) from the trachea of donors with no history of prior lung disease, and the cells were seeded on pre-collagen-coated transwells. Once the cells formed a confluent monolayer (~ 5 days), media was removed from the top chamber the surface of the cells was exposed to air, and the ALI condition was initiated. After 21 days of ALI culture, when they closely resemble the airway epithelial barrier of the cartilaginous airways and are well differentiated with ciliated, mucus, and basal cells and an intact innate immune response, we infected them with the SARS-CoV-2 virus.

We compared the 96-TW ALI (Fig. 1B) with the current gold standard 24-TW ALI (Fig. 1C). We found 96-TW ALI cultures closely resembled the airway epithelial barrier of the mucociliary airways and are well differentiated with (i) uniform nuclei distribution, (ii) ciliated cells, (iii) mucus cells and (iv) basal cells, and have no difference in the distribution of these cells when compared with the 24-TW ALI (Fig. 1B, Ci–iv). We also measured trans-epithelial electrical resistance (TEER) as a measure of tight junction formation of the ALI epithelial barrier, and the week-wise values for both 24-TW- and 96-TW-ALI were within the acceptable range [34] and not significantly different at days 15 and 25 of culture (Fig. 1D). All these data suggest that the 96-TW human ALI model is a relevant model of the mucociliary epithelium and can replace the 24-TW human ALI model to achieve higher throughput.

We identified a few key factors for generating a successful image-grade 96-TW ALI with uniform SARS-CoV-2 infection quality. Firstly, the transwell membrane material is important for imaging. Although human ABSCs grow well on both polycarbonate (PC) and polyester (PET) membranes, only PET membranes are transparent, allowing much higher quality image acquisition during high throughput confocal microscopy. Figure 1Ei–iv demonstrates a typical cluster of SARS-CoV-2 infection in ciliated cells in our PET-based 96-TW ALI, proving its suitability for high-content imaging. Second, we found that the number of cells seeded is essential. We observed that the optimal cell seeding density is a function of active ABSCs in the isolated population. So, expanding the isolated ABSCs up to passage 2 (P2) results in activation and enrichment of the ABSC population and requires less initial cell numbers to obtain a confluent monolayer (10,000 cells/well for unexpanded ABSCs vs. 8000 cells/well for expanded ABSCs), and less time to reach confluency of ABSCs (7 days for unexpanded vs. 5 days for expanded). Apart from cell density and submerged culture duration, other quality parameters were unaltered between P0 and P2, whereas expanding the cell beyond P2 resulted in poor culture quality with uneven cell type distribution and the absence of tight junction formation due to a reduction in ABSC numbers. Thirdly, we identified donor heterogeneity and the need to pool donor cells as key for reproducible drug screening. For this study, we used eight airway donor lung (DL) samples, and we noticed a significant degree of variation in the percentage of infected cells from donor to donor (Fig. 1F). The variation seen in SARS-CoV-2 infection of ALI cultures from different donors is likely due to differences in the host’s innate immune response. Even though recapitulation of this heterogeneity of infection in our model reveals its suitability for personalized medicine, we needed to have a reliable, reproducible response to find a hit compound that has efficacy for a large patient population. Therefore, we pooled ABSCs from three different patients to generate ALI cultures. We followed hepatocyte studies where pooling of cells from different patients is standard practice in drug screening [35]. The demographics of all the ABSC donors used for this study are elucidated in Table 1.

Another crucial parameter for high throughput drug screening is a fast readout with quantification. For our high-content screening, we scanned 14 sites in each well of the 96-TW ALI and built an adaptive thresholding-based algorithm to quantify infection based on the number and intensity of clusters across all sites. For this, we selected infection cluster count/well as our preferred read-out as it was more consistent and allowed validation with ImageJ-based counting. The detailed methods for the quantification algorithm are provided in the methods section.

Once the screen was developed and validated for SARS-CoV-2 infection, we initially infected the ALI cultures with the Wuhan parental SARS-CoV-2 strain (Isolate USA-WA1/2020, BEI Resources NR-52281) at a low MOI (0.1), followed by the Beta (Isolate hCoV-19/USA/MD-HP01542/2021, BEI Resources NR-55282), Delta (Isolate hCoV-19/USA/MD-HP05647/2021, BEI Resources NR-55672), and Omicron (Isolate hCoV-19/USA/MD-HP20874/2021, BEI Resources NR-56461) variants as they arose during the pandemic. Figure 1G and Hi–v demonstrate the variability of infection among these strains in the 96-TW ALI and the corresponding quantification. The mock (no infection) well had no interfering signal or noise (Fig. 1G, Hi). The parental strain had the lowest level of infected clusters per well of 96-TW ALI (99 ± 15) (Fig. 1G, Hii), while the Beta variant had an average of 120 ± 21 (Fig. 1G, Hiii). Delta showed the highest level of infectivity with an average number of 1876 ± 102 infection clusters (Fig. 1G, Hiv), while the Omicron variant showed 280 ± 23 infected clusters per well (Fig. 1G, Hv). The different degrees of infectivity across the SARS-CoV-2 variants appeared to be associated with the known degree of disease severity. We noted differences across viral variants, with some having many small-sized infection clusters, suggesting that entry is easier than replication for that variant. For other variants, there were large clusters of infection that were few in number, suggesting entry was difficult, but replication was easier for that variant. The SARS-CoV-2 infected ALI cultures are, therefore, a potential model to predict response to therapies that are currently in the clinic and to screen for novel compounds. Therefore, we next tested our model for drug screening in HTS format (Fig. 1I).

### Drug screening using the 96-TW ALI model of SARS-CoV-2 infection

**Fig. 2:**
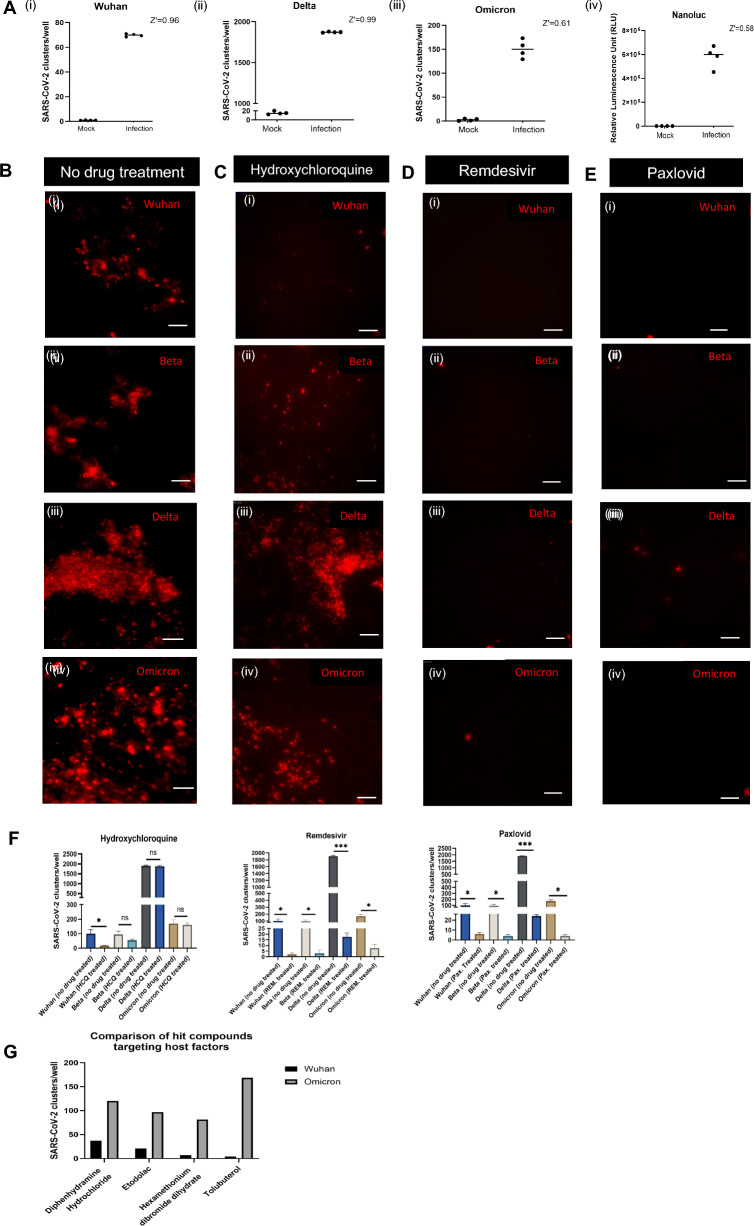
Validation and testing of the 96-TW ALI as a drug screening platform. **A** Z’ values across successive SARS-CoV-2 Strain infections: (i) Wuhan, (ii) Delta, (iii) Omicron, and in the (iv) Wuhan-Nanoluciferase (Nanoluc) reporter assay. **B** IF staining showing degree of infection with no drug treatment across different strains: (i) Wuhan (Spike antibody), (ii) Beta (Spike antibody), (iii) Delta (Nucleocapsid antibody), (iv) Omicron (Nucleocapsid antibody). Scale bar = 50 µm. **C** IF staining showing efficacy of Hydroxychloroquine across different strains: (i) Wuhan (Spike antibody), (ii) Beta (Spike antibody), (iii) Delta (Nucleocapsid antibody), (iv) Omicron (Nucleocapsid antibody). Scale bar = 50 µm. **D.** IF staining showing efficacy of Remdesivir across different strains: (i) Wuhan (Spike antibody), (ii) Beta (Spike antibody), (iii) Delta (Nucleocapsid antibody), (iv) Omicron (Nucleocapsid antibody). Scale bar = 50 µm. **E** Efficacy of Paxlovid against (i) Wuhan (Spike antibody), (ii) Beta (Spike antibody), (iii) Delta (Nucleocapsid antibody), (iv) Omicron (Nucleocapsid antibody). Scale bar = 50 µm. **F** Quantification of SARS-CoV-2 clusters/well among Hydroxychloroquine, Remdesivir, and Paxlovid treated vs. corresponding no drug-treated controls for each strain. P values are calculated from technical replicates using Unpaired t-test between drug-treated and not treated for each strain. *P < 0.05, **P < 0.01, ***P < 0.001. **G** Top hits from the drug screen libraries detailed in Table 1, showing differences in efficacy in 96-TW ALI cultures infected with Wuhan versus Omicron SARS-CoV-2 variants

To validate our 96-TW ALI drug screening platform, we calculated the Z-factor (Z’) [33] of the 96-TW ALI model to test its efficacy for high throughput drug screening (HTS) for SARS-CoV-2 from mock (no virus) and infection (virus only, no drug) based on cluster count. The calculated average Z’ for each infection (Wuhan 0.967, Delta 0.995, Omicron 0.614) proved its suitability for screening for novel drugs for SARS-CoV-2 (Fig. 2A). Beta did not show a robust and consistent Z’ due to higher variability (data not shown). In parallel to the image-based quantification, we also tested and validated the SARS-CoV-2 Wuhan virus reporter NanoLuc Luciferase (Nano-Glo Luciferase assay, Promega) in our system (average Z’ Nanoluc 0.588) (Fig. 2Aiv) for rapid drug screening and quantification purposes.

Next, we examined the degree of infection (number of SARS-CoV-2 clusters) of the SARS-CoV-2 parental strain and variants in the 96-TW ALI model in the presence of the drugs that are in the clinic (Hydroxychloroquine, Remdesivir, and Paxlovid). Compared with no drug-added control (Fig. 2Bi–iv) hydroxychloroquine (HCQ) (10 µM) (Fig. 2Ci–iv) showed no significant effect except for the parental strain Wuhan, where the number of SARS-CoV-2 clusters were reduced significantly (P < 0.05) (Fig. 2F). Unlike hydroxychloroquine, Remdesivir (10 µM) and Paxlovid (Nirmatrelvir-Ritonavir, 10 µM) significantly reduced the number of SARS-CoV-2 clusters in all SARS-CoV-2 strains in our 96-TW ALI model (Fig. 2D–F).

For drug screening in 96-TW ALI, we tested 2100 compounds across 3 libraries [440 from LOPAC (Sigma Aldrich), 660 from New Prestwick (Greenpharma), 1000 from NKIL (Selleck Chemicals)] in 8 airway donor lung (DL) samples, against Wuhan, Beta and Delta and found a total of 72 compounds that reduced infection, elucidating a 2.8% hit rate. Notably, we included 13 NKIL hits (CH5132799, Enzastaurin, AG-490, GDC-0980, Hesperadin, CEP 32496, PP242, AS-604805, AZD8055, IPI-145, URMC-099, JNJ 38877605, AZD2014) targeting cell stress-related signaling pathways, that we previously identified when screening with Vero cells [20, 33], and they were not effective in the human 96-TW ALI model. This demonstrates that screening with an airway epithelium with innate immunity can produce very different results to screening with Vero cells that have no immunity against infection. We also found that the hit compounds were different for different variants, indicating that each variant's inhibition mechanism may be different. All 72 hit compounds from our primary screen were re-screened against Wuhan in one 96-TW ALI culture. Only four compounds—Diphenhydramine, Etodolac, Hexamethonium dibromide dihydrate, and Tulobutarol were effective (average no of COVID-19 clusters/well: 13.33) as they reduced infection by ~ sevenfold compared to no drug control (99 clusters/well) (Fig. 2G). However, when tested against Omicron, they were not nearly as effective (average no of COVID-19 clusters/well: 116.66) (Fig. 2G). Our 96-TW ALI drug screen did not identify any compounds that were effective against both the Wuhan parental strain and the Omicron variant. However, the most effective drugs in our assay were Paxlovid and Remdesivir, both direct-acting antivirals. We therefore collaborated with Merck KGaA, Darmstadt, Germany, to test some of their compounds in our 96-TW ALI screening platform.

### Screening with 96-TW ALI cultures validates a new compound with antiviral defense properties

**Fig. 3:**
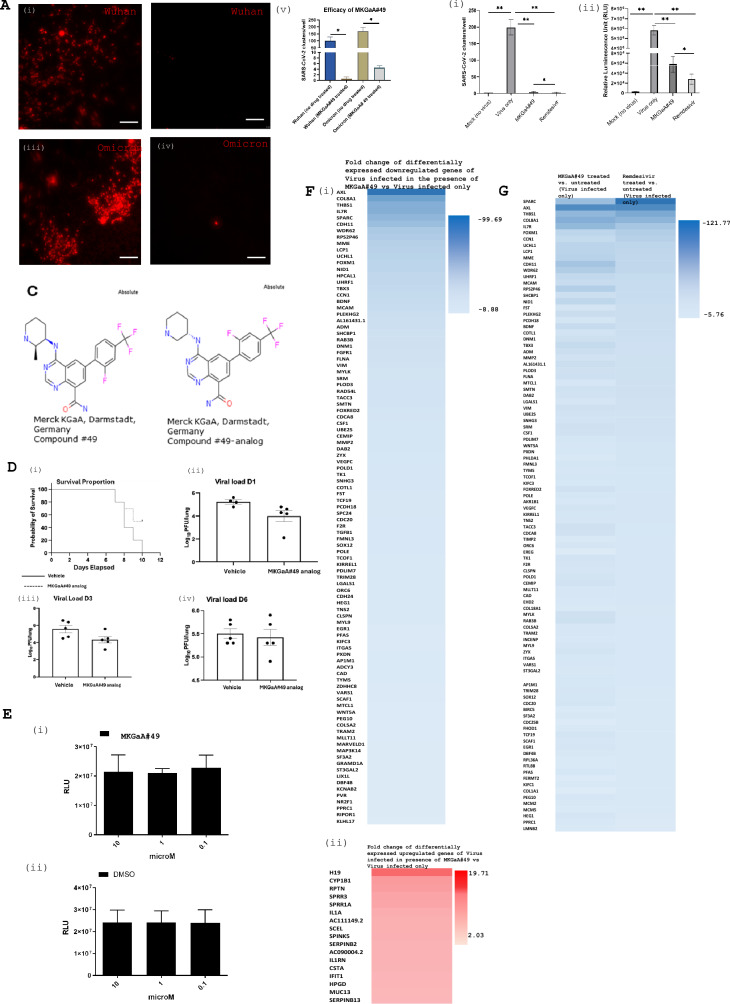
The screen identifies a compound with antiviral efficacy against SARS-CoV-2. **A** IF staining showing the efficacy of MKGaA#49 (Merck KGaA, Darmstadt, Germany Compound#49) in the 96-TW ALI cultures: (i) Wuhan infection only, (ii) Wuhan infection in presence of MKGaA#49, (iii) Omicron infection only, (iv) Omicron infection in presence of MKGaA#49. Scale bar = 50 µm. (v) Quantification of MKGaA#49 efficacy. P values are calculated from technical replicates using Unpaired t-test between drug treated and not treated for each strain. *P < 0.05. **B** Comparative efficacy of MKGaA#49 and Remdesivir in reducing Omicron infection by (i) SARS-CoV-2 infection cluster/well by IF imaging quantification (nucleocapsid antibody), and (ii) Nanoluc assay. P values are calculated from technical replicates using one way ANOVA test. **P < 0.01, *P < 0.05. **C** Structure of MKGaA#49 and a MKGaA#49analog. **D.** In vivo antiviral efficacy of MKGaA#49 analog: (i) Mice exhibited increased survival upon drug treatment. (ii) Mice exhibited reduced viral load at Day 1 post-infection, and (iii) Day 3 post-infection. There was no difference seen in viral load at (iv) Day 6 post-infection. **E** Cell Titre Glo assay to assess for any toxicity of (i) MKGaA#49 across three concentrations as compared to (ii) DMSO alone across the same concentrations. **F** Global RNA sequencing of SARS-CoV-2 infection in presence of MKGaA#49 vs SARS-CoV-2 infection only. Heat map of (i) top 100 downregulated genes and (ii) all 17 upregulated genes. **G** Global RNA sequencing of SARS-CoV-2 infection in presence of MKGaA#49 compared to SARS-CoV-2 infection in presence of Remdesivir. Heat map of top 100 genes differentially expressed in SARS-CoV-2 infection only vs SARS-CoV-2 infection treated with MKGaA#49 compared to the top 100 genes differentially expressed in SARS-CoV-2 infection only vs SARS-CoV-2 infection treated with Remdesivir

In collaboration with Merck KGaA, Darmstadt, Germany, we tested a custom library of 75 small molecules in our 96-TW ALI cultures. We singled out one small molecule (Merck KGaA, Darmstadt, Germany Compound#49, abbreviated as MKGaA#49 henceforth) that showed a significant reduction in infection against the parental strain (Fig. 3Ai, ii) and the Omicron variant (Fig. 3Aiii, iv) resulting in ~ 90% and 62% reduction in infection for Wuhan parental strain and Omicron, respectively (Fig. 3Av). When compared with Remdesivir, MKGaA#49 showed comparable efficacy against Omicron (average no of COVID-19 clusters/well for MKGaA#49: 4.6 vs. Remdesivir: 2.4) (Fig. 3Bi) and in the Wuhan Nanoluc reporter assay (Fig. 3Bii).

The structure of MKGaA#49 (6-(2-Fluoro-4-trifluoromethyl-phenyl)-4-((2R,3R)-2-methyl-piperidin-3-ylamino)-quinazoline-8-carboxylic acid amide) and its close analog: MKGaA#49analog (6-(2-Fluoro-4-trifluoromethyl-phenyl)-4-((2R,3R)-2-methyl-piperidin-3-ylamino)-quinazoline-8-carboxylic acid amide) are shown in Fig. 3C.

We also evaluated the MKGaA#49-analog for in vivo activity in a mouse model. As shown in Fig. 3D, a prolonged survival could be observed in mice treated with the compound compared to vehicle control (Fig. 3Di). Oral treatment of mice with the compound MKGaA#49 resulted in reduced viral titers in the lungs on Day 1 (D1) (Fig. 3Dii), D3 (Fig. 3Diii), and D6 (Fig. 3Div). However, these effects were not statistically significant. When tested with the Cell Titre Glo assay, MKGaA#49 showed very low toxicity, and the results were essentially the same as for the solvent DMSO alone (Fig. 3Ei, ii).

Given the compound’s efficacy both in vitro and in vivo, next we sought to understand its mechanism of action, and we therefore assessed the effect of MKGaA#49 treatment compared to DMSO treatment in SARS-CoV-2 infected 96-TW ALI cultures by global RNA sequencing. When compared differentially expressed genes between the virus infected only and the virus infected in the presence of MKGaA#49, we found many genes that were downregulated by MKGaA#49 in infected ALI cultures and only about 50 upregulated genes (Fig. 3F). In addition to comparing MKGaA#49 with no drug-treated control, we also compared MKGaA#49 with Remdesivir, which showed similar gene expression changes (Fig. 3G). Analysis of the GO terms for these differentially expressed genes showed the antiviral response and innate immune response were induced by MKGaA#49, in addition to negative regulation of viral genome replication (Table 2).

**Table 2:** Functional classification of differentially expressed genes between the virus (Wuhan) infected only and the virus infected in the presence of MKGaA#49

Annotation cluster 1	Enrichment Score: 9.7	Count	P_Values	Benjamini
UP_KW_BIOLOGICAL_PROCESS	Antiviral defense	13	1.10E−15	2.70E−14
GOTERM_BP_DIRECT	Defense response to virus	14	2.00E−15	7.70E−13
GOTERM_BP_DIRECT	Response to virus	11	3.80E−14	7.50E−12
GOTERM_BP_DIRECT	Negative regulation of viral genome replication	9	7.30E−14	9.60E−12
UP_KW_BIOLOGICAL_PROCESS	Innate immunity	13	4.50E−10	5.60E−09
UP_KW_BIOLOGICAL_PROCESS	Immunity	14	5.30E−07	4.40E−06
GOTERM_BP_DIRECT	Innate immune response	10	6.90E−06	5.40E−04
GOTERM_MF_DIRECT	RNA binding	6	2.80E−01	1.00E + 00

Annotation cluster 2	Enrichment score: 5.63	Count	P_Values	Benjamini
KEGG_PATHWAY	Influenza A	8	2.00E−07	1.60E−05
KEGG_PATHWAY	Hepatitis C	7	2.60E−06	1.00E−04
KEGG_PATHWAY	Coronavirus disease—COVID-19	7	2.40E−05	6.40E−04

Annotation cluster 3	Enrichment score: 3.72	Count	P_Values	Benjamini
GOTERM_CC_DIRECT	Cornified envelope	7	4.10E−09	2.70E−07
GOTERM_BP_DIRECT	Keratinocyte differentiation	6	3.20E−07	3.20E−05
INTERPRO	Cornifin (SPRR1)	3	1.50E−05	1.70E−03
GOTERM_BP_DIRECT	Epidermis development	5	3.70E−05	2.20E−03
GOTERM_BP_DIRECT	Peptide cross-linking	4	4.00E−05	2.20E−03
GOTERM_BP_DIRECT	Keratinization	5	4.70E−05	2.30E−03

## Discussion

Here, we have developed a primary human airway cell-based 96-TW ALI model as a drug screening platform for SARS-CoV-2 variants. The 96-TW ALI recapitulated the heterogeneity of infection seen clinically among people infected with SARS-CoV-2 and across infections with different SARS-CoV-2 variants. 96-TW ALI drug screening of FDA-approved and re-purposed drug libraries targeting host factors across variants revealed no consistent hits. However, direct-acting antivirals like Remdesivir and Paxlovid were consistently effective in our model against multiple SARS-CoV-2 variants. When screened against a panel of compounds from Merck KGaA, Darmstadt, Germany, our model identified a novel compound (MKGaA#49) that showed similar efficacy to Remdesivir against the SARS-CoV-2 parental strain (Wuhan) and the Omicron variant. It also showed gene expression differences suggesting antiviral defense, induction of the innate immune response, and inhibition of viral genome replication as its mechanism of action.

Overall, this study highlights a relevant, novel, human drug screening platform for SARS-CoV-2 drug discovery. The study also demonstrates how donor diversity and SARS-CoV-2 variants play a key role in therapeutic discovery and how pooling donors can improve assay variability. An important aspect of our model is that it has innate immunity and, therefore, more closely recapitulates respiratory viral infections of the human airway. Thus, our 96-TW ALI model also has the potential to be used for drug discovery for other respiratory viral infections. While ALI cultures have been used for decades, they have not been used for moderate to high throughput drug screening for viral infections because throughput has been lacking. This is because high throughput imaging and quantification have been difficult to achieve.

## Conclusions

Here we show how we have overcome the barriers to screening of the SARS-CoV-2 infected primary human mucociliary airway epithelium and validated MKGaA#49, which shows potential for treating multiple variants of COVID-19 and possibly other respiratory viruses. Our data also demonstrates that compounds targeting host factors from FDA-approved and repurposed drug libraries show variability in response across donors. At the same time, direct-acting antivirals were effective against all donors and variants. This suggests that the direct-acting antiviral chemical space should be expanded and mined with assays such as ours to identify the next generation of therapies for respiratory viral infections and possible future pandemics.

### Limitations of the study

While we speculate that the throughput could be advanced even further on our 96-TW ALI culture model, we could only achieve moderate throughput with our available technology. We speculate that as high-throughput confocal imaging technologies develop, our 96-TW ALI culture model will become higher throughput, and the quantification algorithms will become more sophisticated. We were also limited in the age range of airway donor tissue we received, so we did not study variability in infection in young children compared to older adults.

## Data Availability

Sequence data that support the findings of this study have been deposited in GEO with the accession number GSE266907. To review GEO accession GSE266907: Go to https://www.ncbi.nlm.nih.gov/geo/query/acc.cgi. Enter token wlqruamqjlyfler into the box.
